# Suppression of porcine hemagglutinating encephalomyelitis virus replication by resveratrol

**DOI:** 10.1186/s12985-022-01953-5

**Published:** 2022-12-28

**Authors:** Yuzhu Liu, Deguang Song, Xueli Liu, Yuanqi Wang, Gaili Wang, Yungang Lan

**Affiliations:** 1 Key Laboratory of Zoonosis Research, Ministry of Education, College of Veterinary Medicine, Jilin University, Changchun, China; 2 Jilin Academy of Animal Husbandry and Veterinary Medicine, Changchun, Jilin China

**Keywords:** Porcine hemagglutinating encephalomyelitis virus, Resveratrol, Antiviral activity, *Betacoronavirus*

## Abstract

**Background:**

Porcine hemagglutinating encephalomyelitis virus (PHEV), a member of the genus *Betacoronavirus*, is the causative agent of neurological disease in pigs. No effective therapeutics are currently available for PHEV infection. Resveratrol has been shown to exert neuroprotective and antiviral effects. Here resveratrol was investigated for its ability to inhibit PHEV replication in nerve cells and central nervous system tissues.

**Methods:**

Anti-PHEV effect of resveratrol was evaluated using an in vitro cell-based PHEV infection model and employing a mouse PHEV infection model. The collected cells or tissues were used for quantitative PCR analysis, western blot analysis, or indirect immunofluorescence assay. The supernatants were collected to quantify viral loads by TCID_50_ assay in vitro. EC50 and CC50 were determined by dose–response experiments, and the ratio (EC50/CC50) was used as a selectivity index (SI) to measure the antiviral versus cytotoxic activity.

**Results:**

Our results showed that resveratrol treatment reduced PHEV titer in a dose-dependent manner, with a 50% inhibition concentration of 6.24 μM. A reduction of > 70% of viral protein expression and mRNA copy number and a 19-fold reduction of virus titer were achieved when infected cells were treated with 10 µM resveratrol in a pre-treatment assay. Quantitative PCR analysis and TCID_50_ assay results revealed that the addition of 10 μM resveratrol to cells after adsorption of PHEV significantly reduced 56% PHEV mRNA copy number and eightfold virus titer. 10 µM resveratrol treatment reduced 46% PHEV mRNA copy number and fourfold virus titer in virus inactivation assay. Moreover, the in vivo data obtained in this work also demonstrated that resveratrol inhibited PHEV replication, and anti-PHEV activities of resveratrol treatment via intranasal installation displayed better than oral gavage.

**Conclusion:**

These results indicated that resveratrol exerted antiviral effects under various drug treatment and virus infection conditions in vitro and holds promise as a treatment for PHEV infection in vivo.

**Supplementary Information:**

The online version contains supplementary material available at 10.1186/s12985-022-01953-5.

## Introduction

Porcine hemagglutinating encephalomyelitis virus (PHEV) is a member of the genus *Betacoronavirus* (βCoV), the family Coronaviridae. PHEV invades the central nervous system (CNS) via the peripheral nervous system and causes porcine hemagglutinating encephalomyelitis (PHE) in suckling piglets [1–4]. The high prevalence of PHE, one of the most common viral diseases in pigs worldwide, stems from high rates of subclinical PHEV transmission occurring within most swine herds [3, 5, 6]. Nevertheless, this disease is not clinically relevant in most swine-producing regions because dams are immune to PHEV infection and thus provide passive immunity to their offspring [5, 7, 8]. However, after PHEV-infected pigs that survive the disease recover from acute PHE-associated immunopathology, negative effects of the disease on animal growth become apparent [7, 9, 10]. Recently, increased research attention has been being focused on PHE, due to viral evolution in some countries and high mortality rates on pig farms with certain breeds of pathogen-free animals [6, 8, 11–13]. Although swine species are the only animals known to be naturally susceptible to PHEV natural infection, laboratory rodents (e.g., mice) have served as alternative animal models for investigations of PHEV pathogenesis, due to similarities between PHEV infections of pigs and mice [14, 15]. Meanwhile, researchers have identified several underlying mechanisms for PHE pathogenesis by using mice as model systems, but these efforts have not yet led to the development of effective treatments for PHEV infection [16].

Resveratrol (3,5,4′-trihydroxystilbene, RES) is a nonflavonoid polyphenol, which is found in various types of fruits (*e.g., grapes berries*) and roots of herbal traditional Chinese medicines (*e.g., Pediomelum cuspidatum*) [17]. RES has attracted widespread attention, due to its demonstrated efficacy for preventing and alleviating neurological disorders as reported in studies conducted using animal models and models based on cells cultured in vitro [18–20]. RES is being extensively studied in the amelioration of multiple βCoVs such as Severe acute respiratory syndrome coronavirus 2 (SARS-CoV-2) and Middle east respiratory syndrome coronavirus (MERS-CoV) infections in vitro [21, 22]. Moreover, RES has been reported to have antiviral activity against Pseudorabies virus (PRV) and other neurotropic viruses both in vitro and in vivo, with RES antiviral effects shown to be associated with anti-inflammatory and antioxidant activities of the compound [23, 24]. However, the antiviral effect of RES on neurotropic PHEV replication is still unclear.

PHEV targets nervous system neurons by entering and replicating in nerve cells, prompting researchers to employ PHEV-permissive mouse neuroblastoma (N2a) cells or mice as model systems to study CNS pathology associated with PHEV-induced encephalitis [25]. In the present study, N2a cells and mouse PHEV-infection models were used to evaluate RES for anti-PHEV effects. These results thus warrant the development of drugs to prevent and treat PHE.

## Material and methods

### Virus, cells, and reagents

The PHEV Stain used in this study was PHEV CC14 (GenBank accession number MF083115.1), which was maintained by our research group [26]. N2a cells were supplied by ATCC (Manassas, VA, USA). RES (C_14_H_12_O_3_) of 98% purity was purchased from Sigma-Aldrich (St-Louis, MO, USA) and was dissolved into dimethyl sulfoxide (DMSO; Sigma-Aldrich, St-Louis, MO, USA) at 100 mM and stored at − 20 °C. For antiviral assays, RES was diluted in Dulbecco's Modified Eagle Medium (DMEM; Meilunbio, Dalian, CN) containing 2% fetal bovine serum (FBS; BI, Kibbutz Beit Haemek, Israel) to create RES solutions of various concentrations. DMSO + DMEM solution served as the solvent control. Mouse anti-PHEV-Nucleocapsid (N) protein monoclonal antibody was prepared in our laboratory. Alexa Fluor 488-labeled donkey, anti-rabbit secondary, and Hoechst 33,342 were obtained from Thermo Fisher Scientific (Waltham, MA, USA).

### ***Virus propagation and the viral 50% tissue culture infectious dose (TCID***_***50***_***) assays***

Briefly, the N2a cells were propagated in DMEM supplemented with 2% FBS and 1% antibiotic–antimycotic (Gibco, Grand Island, NY, USA). A monolayer of N2a cells was washed twice with phosphate-buffered saline (PBS) (0.01 M, pH 7.4), and then inoculated with virus suspension. After adsorption for 1 hour (h) at 37 °C in a humidified atmosphere with 5% CO2, the cells were washed 3 times with PBS, and 2% DMEM was added. The cell cultures were examined daily for cytopathic effect (CPE). When more than 80% CPE was evident in the inoculated cell monolayers, the cells and supernatants were harvested together, subjected to three freeze–thaw cycles, serially diluted tenfold from 10^−1^ to 10^−8^, and added to N2a cells in 96-well plates. After 3 days of infection, the TCID_50_ was calculated using the Spearman-Kärber method [27, 28].

### Cytotoxicity assay

Cytotoxicity of RES was measured using a commercial CCK-8 assay kit (Beyotime, Nantong, Jiangsu, CN). Briefly, N2a cells were cultured in 96-well plates at 4 × 10^4^ cells per well at 37 °C in a humidified atmosphere with 5% CO2. After a 12 h incubation, each group of cells was treated with or without various concentrations of RES (10, 25, 50, 100, and 150 μM) at 37 °C with 5% CO2 for 24 or 48 h. Next, 10 μL of CCK-8 assay solution was added to each well then plates were incubated for an additional hour. Next, absorbances of wells were measured at a wavelength of 450 nm. All data are representative of at least three independent experiments. The relative cell viability rate was calculated and expressed as a percentage for each RES concentration. The 50% cytotoxic concentration (CC50), defined as the RES concentration that reduced cell viability by 50%, was calculated based on a non-linear regression model fitted to data collected at 24 and 48 h post-infection (pi).

### Effect of RES on the viability of PHEV-infected N2a cells

N2a cell monolayers in 96-well plates were infected with PHEV at 50 μL suspension that was 100 TCID_50_ (TCID_50_ = 10^–6.125^/0.1 mL). After adsorption for 1 h at 37 °C with 5% CO2, the virus suspension was removed and washed 3 times with PBS (0.01 M, pH 7.4). Next, serial two-fold dilutions of RES (2.5, 5, 10, and 20 μM) were added to the cells then the cultures were incubated at 37 °C with 5% CO2 overnight. When 70% of infected cells exhibited signs of a CPE, cell viability was assessed via CCK-8 assay, and supernatants were collected and tested via TCID_50_ assay to quantify viral loads, as previously described [27, 28]. DMSO + DMEM solution served as the solvent control. All data are representative of at least three independent experiments. Inhibition rate (%) was calculated following the formula: Inhibition rate % = (cell viability of RES-treated PHEV-infected cells—cell viability of the untreated PHEV-infected group/cell viability of the control cells-cell viability of RES-treated PHEV-infected cells) × 100% [29]. RES 50% effective concentration (EC50) values corresponding to 50% inhibition of viral replication were calculated based on a non-linear regression model fitted to the data. The selectivity index (SI) was defined as the ratio of CC50 to EC50.

### Influence of RES on the viral growth curve

N2a cells grown in 6-well plates were infected with PHEV at 200 μL suspension that was 100 TCID_50_ (TCID_50_ = 10^–6.125^/0.1 mL) in the presence or absence of 10 μM RES. After adsorption for 1 h at 37 °C with 5% CO2, the virus suspension was removed and washed 3 times with PBS, and then a maintenance medium containing 10 μM RES was added to the cells. Next, total RNA was extracted from supernatants of infected cells at time points of 6, 12, 24, 32, and 48 hpi then numbers of copies of the PHEV N gene were determined using the Real-Time Quantitative PCR (qPCR) analysis. All data are representative of at least three independent experiments.

### Experimental design

Three protocols were used to investigate the inhibitory effect of RES on PHEV replication as follows [30]: (1) Pre-treatment assay: A monolayer of N2a cells in 24-wells plat was pre-treated with 10 μM RES for 12 h at 37 °C with 5% CO2 before viral adsorption. Next, the culture medium containing the drug was removed then cells were inoculated with 100 μL of PHEV solution that was 100 TCID_50_ (TCID_50_ = 10^–6.125^/0.1 mL) for 24 h at 37 °C with 5% CO2. (2) Replication assay: after N2a cells were infected with 100 μL of PHEV solution that was 100 TCID_50_ (TCID_50_ = 10^–6.125^/0.1 mL) for 4 h at 37 °C with 5% CO2, the PHEV solution was removed and washed 3 times with PBS, and then maintenance medium containing 10 μM RES was added to the cells followed by incubation of cultures for 24 h at 37 °C with 5% CO2. (3) Virus inactivation assay: the suspension containing 100 TCID_50_ (TCID_50_ = 10^–6.125^/0.1 mL) of PHEV was incubated with 10 μM RES for 2 h at room temperature, then the PHEV-RES mixture was added to N2a cells followed by incubation for 1 h at 37 °C with 5% CO2. Next, supernatants containing the virus inoculum were removed by aspiration, a maintenance medium was added to cells, then cultures were incubated for 24 h at 37 °C with 5% CO2. In addition, collected cells were used for qPCR analysis, Western blot analysis, or Indirect immunofluorescence assay (IFA). Meanwhile, supernatants were collected to quantify viral loads by TCID_50_ assay using protocols (1), (2), and (3). All data are representative of at least three independent experiments.

### Animal experiments

Six-week-old BALB/c mice (male) were obtained from the Laboratory Animal Centre of Jilin University. RES was dissolved in 0.5% sodium carboxymethyl cellulose solution (CMC) diluted in saline. Mice were randomly divided into 4 groups (n = 6 per group) as follows: (1) PHEV-infected + treated with 0.5% CMC by oral gavage daily; (2) PHEV-infected + treated with 0.5% CMC by intranasal instillation daily; (3) PHEV-infected + treated with 50 mg/kg of RES followed by administration of 50 μl of different concentrations of RES by instilling intranasally daily; (4) PHEV-infected + treated with 50 mg/kg of RES followed by administration of 1 mL of different concentrations of RES by oral gavage daily [31–33]. Mice were treated with RES for 7 days before PHEV infection then mice of each group continued to receive the same dosages of the experimental drug for 7 days. Next, the mice were anesthetized with isoflurane and then intranasally inoculated with 100 μL of PHEV solution containing 50 TCID_50_ (TCID_50_ = 10^–6.125^/0.1 mL). The single RES treatment via the intranasal installation or oral gavage group served as a negative control. The body weight of mice in each group was monitored after PHEV infection. Mice were euthanized at 7 days post-infection (dpi) by CO2 inhalation according to animal handling guidelines. After sacrifice, the brain was isolated and placed on an ice pad, then the collected brain was used for qPCR analysis, Western blot analysis, or IFA.

### Western blot analysis

Western blot analysis was performed as described previously [26]. Briefly, cells or tissues were lysed in RIPA buffer then lysates were loaded onto SDS-PAGE gels and subjected to electrophoresis. Next, separated proteins in gels were transferred to PVDF membranes (Millipore, Boston, MA, USA) that were probed with appropriate antibodies. Western blot signals were analyzed using Image J software.

### qPCR analysis

For qPCR analysis, RNA was purified from cells or tissues using TRIzol reagent (Thermo Fisher Scientific, Waltham, MA, USA). Two micrograms of total RNA were reverse transcribed using a poly (T) primer and SuperScript III Reverse Transcriptase (Thermo Fisher Scientific, Waltham, MA, USA). PHEV N gene RNA was detected as previously described using primer and probe sequences that targeted the nucleocapsid gene of PHEV [34]. A LightCycler 480 (Roche Applied Science, Indianapolis, IN, USA) was used to perform qPCR. Thermo cycling amplification conditions were as follows: 50 °C for 30 min, 94 °C for 15 min then 45 cycles of 95 °C for 15 s, 58 °C for 60 s, and 72 °C for 10 s, as previously described [34].

### IFA

N2a cells growing on glass coverslips were fixed in 4% paraformaldehyde for 10 min and permeabilized with 0.2% Triton X-100 for 10 min, after which they were blocked with 5% nonfat dry milk (Thermo Fisher Scientific, Waltham, MA, USA) for 1 h at room temperature. The cells were then probed with anti-PHEV-N (1:500) and incubated overnight at 4 °C. After three washes with PBS, the cells were incubated for 1 h at room temperature with Alexa Fluor 488-labeled donkey anti-rabbit secondary antibody at a 500-fold dilution, and then the cell nuclei were labeled with Hoechst 33,342 (1:1,000). Next, cells were washed then coverslips with attached cells were viewed using an Olympus FV1000 confocal microscope (Olympus, Shinjuku-ku, Tokyo, Japan).

### Statistical analysis

Graphical analyses were performed using GraphPad Prism software Version 5.01 (GraphPad Software Inc., La Jolla, CA, USA). In addition, CC50 and EC50 are calculated based on a non-linear regression model fitted to the data by using it. Data are presented as the means ± SD. Analysis of treatment and control group data was performed via Student’s t-test or one-way analysis of variance (ANOVA). *, *P* values < 0.05 were considered statistically significant. **, *P* values < 0.01 were considered statistically very significant. ***, *P* values < 0.001 were considered statistically highly significant.

## Results

### *Evaluation of RES cytotoxicity and antiviral activity against PHEV *in vitro

**Fig. 1:**
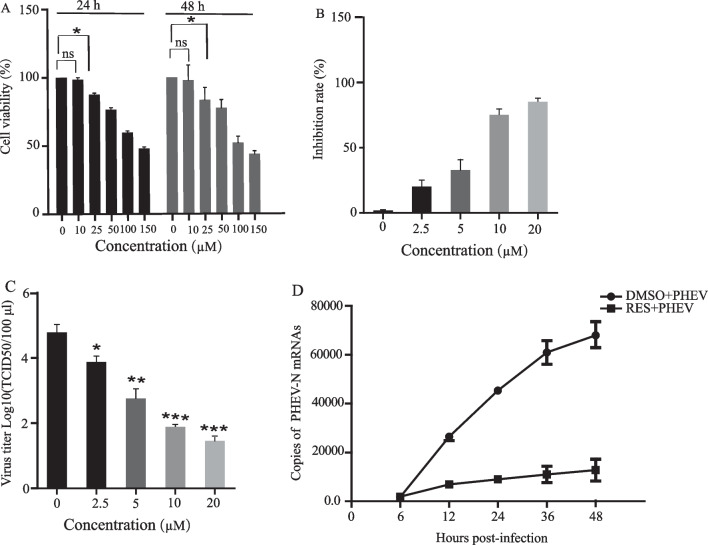
Cytotoxicity and antiviral activity of RES against PHEV in *vitro*. **A** CCK-8 assays were performed on N2a cells exposed to RES for 24 and 48 h to assess the cytotoxicity of RES, respectively. **B** Rate of RES inhibition of PHEV infection: N2a cells were first infected with PHEV and then treated with RES. a CCK-8 assay was performed, and Inhibition rate % was calculated following the formula: Inhibition rate % = (cell viability of RES-treated PHEV-infected cells—cell viability of the untreated PHEV-infected group/cell viability of the control cells-cell viability of RES-treated PHEV-infected cells) × 100%. **C** Viral titers were obtained from PHEV-infected cells treated with different RES concentrations: N2a cells were infected with PHEV and then treated with RES. After cells were cultured for 48 h, supernatants were collected and analyzed by TCID_50_ assay. **D** PHEV replication was monitored in N2a cells in the presence or absence of RES for 48 h, with total RNA extractions performed at indicated time points and viral RNA quantification conducted via qPCR assay. All data are representative of at least three independent experiments. Data are presented as means ± SEM. ns: *p* > 0.05, *p < 0.05, ***p < 0.001 based on comparisons to 0 μM RES-treated cells

To assess RES cytotoxicity, CCK-8 assays were performed to assess N2a cell viability. After treatment of cells with RES for 24 or 48 h at concentrations of 10, 25, 50, 100, and 150 μM, no significant cytotoxicity was observed for RES concentrations < 25 μM (Fig. 1A). The 50% cytotoxic concentration (CC50) was above 139.78 μM, which was calculated based on the data obtained for cells treated with RES for 48 h. To assess RES antiviral efficacy, the 50% effective dose (EC50) of RES in vitro was determined by adding RES directly to PHEV-infected N2a cells using a broad range of RES concentrations (0–20 µM). Next, data were collected after 48 h of RES treatment then the RES EC50 value against PHEV was determined and found to be 6.24 μM and the selectivity index (SI) of RES for PHEV-infected N2a cells was found to be 20.16 (Fig. 1B).

To further evaluate RES antiviral effects, virus yield reduction assays were conducted. The results revealed that PHEV titer in the presence of RES was significantly reduced in a dose-dependent manner with increasing RES concentration. Specifically, the TCID_50_ assay was performed to examine the antiviral effect of RES on the production of viable viruses. The results showed that in the absence of RES treatment, PHEV titer reached 10^5.35^/mL at 48 h post-infection (hpi). By contrast, titers at 48 hpi were 10^3.01^, 10^2.02^, and 10^1.89^/mL for cells treated with 5, 10, and 20 μM RES, respectively, which corresponded to 218-, 2193-, and 2902-fold reductions of virus titers as compared to titers obtained from untreated cells (Fig. 1C).

To determine RES effects on PHEV replication, N2a cells were infected with PHEV in the presence or absence of 10 µM RES. Next, viral genome copy numbers were determined via qPCR analysis based on a standard curve (with an R2 value of 0.9893) using the formula: lg [virus copies] =  − 4.0959Cq + 52.846. PHEV growth curves, as determined from the virus in supernatants of infected cells in the presence or absence of RES, are shown in Fig. 1D. In untreated cells, PHEV began to replicate at 6 hpi then viral N-gene RNA copy numbers rapidly increased over the next 42 h. By contrast, in RES-treated cells almost no viral replication was observed between 6 and 12 hpi then the number of RNA copies increased slowly over the next 36 h but remained significantly lower than the corresponding numbers obtained for untreated cells.

### *Evaluation of RES antiviral activity against PHEV using an *in vitro* cell-based model and different drug treatment and virus infection conditions*

**Fig. 2:**
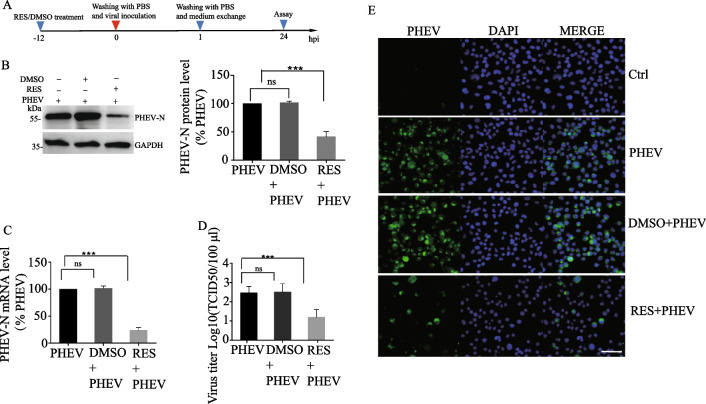
Pre-treatment effect of RES on PHEV replication in N2a cells. **A** Schematic diagrams illustrating the experimental design for time-of-addition experiments. **B**, **C** Expression of PHEV protein and mRNA in N2a cells at 24 h as determined by Western blot and qPCR assay, respectively. PHEV protein and mRNA levels were quantified and normalized to GAPDH levels, respectively. **D** TCID_50_ values were the means of three repeated titrations at the time points indicated. **E** An immunofluorescence assay was performed to examine PHEV expression in N2a cells at 24 h after RES treatment. Labeled and stained cells were viewed using confocal microscopy. PHEV-positive cells were labeled with an anti-N protein monoclonal antibody (green) and nuclei were stained with Hoechst (blue). Scale bar = 100 μm. Data are presented as means ± SEM. *p < 0.05, **p < 0.01, ***p < 0.001 based on comparisons to untreated PHEV-infected cells

**Fig. 3:**
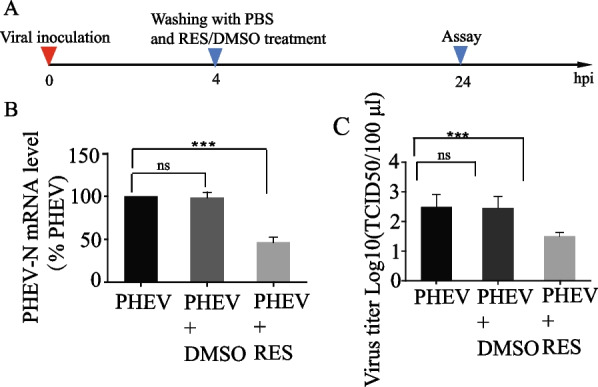
Anti-PHEV effect of RES in replication assay. **A** Schematic diagrams illustrating the experimental design for time-of-addition experiments. **B** Expression of PHEV mRNA in N2a cells at 24 h as determined by qPCR assay. PHEV mRNA levels were quantified and normalized to GAPDH levels, respectively. **C** TCID_50_ values were the means of three repeated titrations at the time points indicated. Data are presented as means ± SEM

We examined the anti-PHEV effect of RES on N2a cells under various treatment conditions. Before PHEV infection (Fig. 2A), it was found that at a concentration of 10 μM RES both PHEV N gene transcription and protein expression levels were > 70% reduction as compared to corresponding control group levels (Fig. 2B, C). In the virus yield reduction assay, the TCID_50_ assay was performed to examine the antiviral effect of RES on the production of viable viruses. The results (Fig. 2D) showed that in the absence of RES, the PHEV titer reached 10^2.47^ TCID_50_/mL at 24 hpi, a titer similar to that obtained from DMSO-treated infected cells. By contrast, the titer at 24 hpi was 10^1.2^ for RES-treated cells, a 19-fold reduction of titer as compared to that obtained from untreated cells. These results were consistent with IFA results, which showed that 10 μM RES significantly inhibited viral replication (Fig. 2E). In addition, qPCR and TCID_50_ assay results revealed that the addition of 10 μM RES to cells after adsorption of PHEV significantly inhibited PHEV replication, with qPCR and TCID_50_ assay results shown in Fig. 3A–C, respectively (56% reduction of PHEV-N mRNA copy number; eightfold reduction of PHEV titer). Moreover, a PHEV suspension was incubated with RES for 2 h. Next, this mixture was added to N2a cells and PHEV infection was allowed to proceed for 1 h then cell cultures were incubated for 24 h. Next, cells were tested via qPCR analysis and TCID_50_ assay. The results showed that 10 µM of RES showed a 46% reduction of PHEV-N mRNA copy number and a fourfold reduction of virus titer as compared to the control group levels (Additional file 1: A–C). Taken together, these results indicated that RES treatment could directly inhibit PHEV replication.

### *RES inhibited PHEV replication *in vivo

**Fig. 4:**
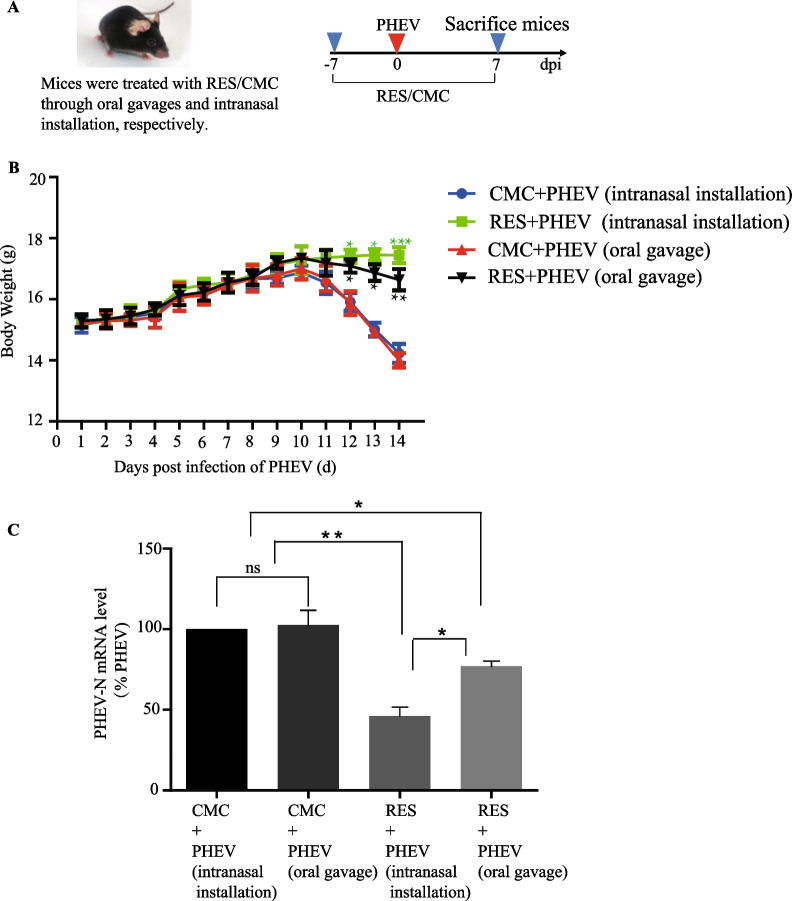
Anti-PHEV efficacy of RES in mice. **A** Standard experimental protocols were used to investigate RES effects in PHEV-infected mice. **B** Changes in body weights of mice in each treatment group were monitored daily. **C** Relative expression levels of viral mRNA in brain tissue at 7 days after PHEV infection were determined via qPCR assay. PHEV mRNA levels were quantified and normalized to GAPDH mRNA levels. N = 6 mice per group. Data are presented as means ± SEM. *P < 0.05, **P < 0.01 versus control-treated groups (PHEV + CMC)

Next, we investigated the effects of RES treatment via intranasal instillation and oral gavage by using PHEV-infected mice as a mouse-based model system of PHE, respectively (Fig. 4A) [14]. Evaluation of changes in body weights of mice after PHEV infection and RES treatment revealed that body weights of RES-treated PHEV-infected BALB/c mice have begun to become significantly heavier on day 11, compared to CMC-treated PHEV-infected mice (Fig. 4B), while both RES treatment via intranasal installation and oral gavage could not inhibit body weights of mice continually loss compared to PHEV-uninfected mice until the end of the observation period (data not shown). To further investigate RES antiviral activity in PHEV-infected mice, viral genomic RNA synthesis levels in brain samples of PHEV-infected mice were measured via qPCR assays. The results revealed that RES treatment of PHEV-infected mice led to a significant reduction of viral genomic RNA synthesis in brain tissues as compared to corresponding levels in CMC-treated PHEV-infected control group mice (PHEV + CMC), and anti-PHEV activities of RES treatment via intranasal instillation displayed better than oral gavage (intranasal instillation, > 50% reduction of PHEV mRNA copy number; oral gavage, > 30% reduction of PHEV mRNA copy number) (Fig. 4C). Taken together, these results preliminarily confirmed that RES treatment of PHEV-infected mice reduced viral replication. However, further research is needed to shed more light on RES effects on PHEV replication by using an in vivo mice-based model.

## Discussion

βCoVs are not always confined to the respiratory tract and most of them may invade the central nervous system (CNS) of humans and/or animals [35], inducing neurological diseases including MERS-CoV [36], Severe acute respiratory syndrome coronavirus (SARS-CoV) [37], Human Coronavirus 229E (HCoV-299E) [38], Human Coronavirus OC43 (HCoV-OC43) [39], SARS-CoV-2 [40], Mouse hepatitis virus (MHV) [41] and PHEV, yet no approved treatments exist that can alleviate infections caused by almost all βCoVs [3, 35, 42]. Importantly, several research studies have used PHEV, also known as porcine neurotropic coronavirus, as a model to study βCoV replication and pathogenic processes with relevance to diseases caused by other βCoVs [26, 43, 44]. The advantages of PHEV as a model are two-fold: PHEV can be handled safely in BSL-2 laboratories while other βCoVs require additional precautions; PHEV can infect multiple cell types of a broad range of host species [3, 44]. Therefore, the development of effective anti-PHEV drugs may be relevant to achieving the prevention and control of diseases caused by other βCoVs.

Potential antiviral drugs against multiple βCoVs must have the ability to enter the CNS in therapeutic amounts, due to the intrinsic neurotropic nature of most βCoVs [35]. PHEV displays neurotropism in mice and Wistar rats and produces acute encephalomyelitis [45, 46], and PHEV infection may be a risk factor for neurodegenerative diseases [26, 47]. In vivo experiments have shown that RES exhibits neuroprotective benefits in animal models of neurodegenerative diseases such as Parkinson’s disease (PD) and Alzheimer’s disease (AD) [48, 49]. Remarkably, evidence obtained from our results conducted by using in vitro or in vivo PHE models indicated that RES treatment could suppress PHEV replication in vitro or in vivo. In addition, we also found that RES treatment protected mouse neuroblastoma cells from cytopathic PHEV infection (Additional file 2). Such results have fuelled speculation that RES can not only interfere with PHEV replication but also reduce the damage to neurons caused by PHEV infection in vivo. The role of RES neuroprotective effect in its anti-PHEV activity warrants further study.

Indeed, the RES has limitations as a treatment for CNS-associated diseases (e.g., PHEV infection), due to its low solubility, instability, and low bioavailability when administered orally [50, 51]. After reaching the bloodstream, RES could be rapidly absorbed by the liver, where it is quickly metabolized and eliminated [50, 52]. A better approach is needed to improve RES delivery to tissues and organs targeted by PHEV. Intranasal administration is an effective interest method of delivering drugs to the CNS, which is non-invasive and allows large molecules that do not cross the blood–brain barrier access to the CNS [53]. In addition, delivery from the nose to the CNS occurs along both the olfactory and trigeminal neural pathways via an extracellular route and does not require the drug to bind to any receptor or axonal transport, which can reduce systemic exposure and thus unwanted systemic side effects [53, 54]. Moreover, the use of intranasal administration of RES might allow for direct delivery of the treatment into the lung. Indeed, intranasal administration of a concentrated formulation has proved to be a successful method to expose the lungs of A/J mice to a sufficient amount of RES [55]. Thus intranasal administration could provide maximum anti-SARS-CoV-2 efficiency, its administration as a nasal spray might be researched for overcoming the poor bioavailability of RES in human clinical trials to fight COVID-19 [21, 56]. In addition, our results also showed that anti-PHEV activities of RES treatment via intranasal installation displayed better than oral gavage in vivo, indicating that intranasal delivery of RES might be a better approach for the treatment of PHE.

The antiviral mechanisms of RES have been widely studied in several viruses [57]. Its main antiviral mechanisms were seen to be elicited by inhibition of various transcription and signaling pathways, inhibition of viral protein synthesis, and inhibition of viral gene expressions [58]. For example, its antioxidant effect is confirmed through the inhibition of important gene pathways like the NF-κβ pathway, while its antiviral effects are associated with inhibitions of gene expression, protein synthesis, nucleic acid synthesis, and viral replication [58, 59]. In addition, it has been reported that autophagy is a powerful tool that host cells use to defend against multiple viral infections, and lysosomes play key roles in host antiviral defenses through virus degradation and modulating the metabolic turnover of proteins related to immune response associated with biological signal pathways [60, 61]. Interestingly, the latest research shows that βCoVs recruit cellular autophagy mechanisms for their replication, and use a lysosome-based egress pathway independent of the biosynthetic secretory pathway, and this potentially opens up new therapeutic avenues to mitigate coronavirus infection and slow virus spread by using drugs as the targeting regulators of autophagy and lysosome function [62, 63]. For example, autophagy is necessary for the replication of PHEV in nerve cells, and PHEV can localize to the lysosomes and lead directly to lysosome dysfunction [26, 64]. RES as an inducer of autophagy can promote autophagy and lysosomal function via ER calcium-dependent TFEB activation [65]. Thus, the autophagy and lysosome-based pathway may also be involved in RES antiviral and neuroprotective effects within the context of infections caused by PHEV, warranting further investigations.

## Conclusions

This is the first study to evaluate the anti-PHEV activity of a natural compound against PHEV. The results of this study demonstrated that RES was effective at inhibiting PHEV replication and was able to block the expression of viral protein and genomic RNA synthesis in vitro. In addition, RES treatment could reduce viral RNA levels within mouse CNS tissues. Indeed, we found that RES when added to PHEV-infected cells exhibited direct virucidal activity against PHEV that increased with increasing RES concentration. Taken together, we confirmed that RES has potential value as a treatment to combat PHEV infection.

## Supplementary Information

**Additional file 1**: N2a cells infected with PHEV were treated with RES Virus inactivation assay. **A** Schematic diagrams illustrating the experimental design for time-of-addition experiments. **B** Expression of PHEV mRNA in N2a cells at 24 h as determined by qPCR assay. PHEV mRNA levels were quantified and normalized to GAPDH levels, respectively. **C** TCID_50_ values were the means of three repeated titrations at the time points indicated. Data are presented as means ± SEM. *p < 0.05, **p < 0.01, ***p < 0.001 based on comparisons to untreated PHEV-infected cells.

**Additional file 2**: N2a cells were pre-treated with 10 μM RES for 12 h at 37 °C with 5% CO2 before viral adsorption. Next, the culture medium containing the drug was removed then cells were inoculated with PHEV at 100 TCID_50_ (10^6.125^) for 72 h at 37 °C with 5% CO2. Mock-infected cells stuck tightly to the plate and remained in good condition throughout the experiment. In contrast, RES treatment effectively blocked CPE in the cell cultures compared to DMSO-treated PHEV-infected cells. **A** Mock-infected cells. **B** RES-treated PHEV-infected cells. **C** DMSO-treated PHEV-infected cells. Cell images were captured by a light microscope. The experiment was performed in triplicate and repeated 3 times. Scale bar = 20 μm.

## Data Availability

All data and materials described in the manuscript are available.

## Abbreviations

PHEV: Porcine hemagglutinating encephalomyelitis virus
PHE: Porcine hemagglutinating encephalomyelitis
RES: Resveratrol
βCoVs: *Betacoronaviruses*
CNS: Central nervous system
SARS-COV-2: Severe acute respiratory syndrome coronavirus 2
MERS-CoV: Middle east respiratory syndrome coronavirus
PRV: Pseudorabies virus
SARS-CoV: Severe acute respiratory syndrome coronavirus
HCoV-299E: Human Coronavirus 229E
HCoV-OC43: Human Coronavirus OC43
MHV: Mouse hepatitis virus
PD: Parkinson’s disease
AD: Alzheimer’s disease
CC50: The 50% cytotoxic concentration
EC50: The 50% effective dose
SI: The selectivity index
QPCR: Quantitative PCR
IFA: Indirect immunofluorescence assay
N2a: Mouse neuroblastoma
TCID_50_: 50% Tissue culture infectious dose
