# Supplementary figures and images for: Suppression of porcine hemagglutinating encephalomyelitis virus replication by resveratrol
Source: Virol J. 2022 Dec 28;19:226. doi: 10.1186/s12985-022-01953-5 (PMC9795454; doi:10.1186/s12985-022-01953-5)

Additional File 1

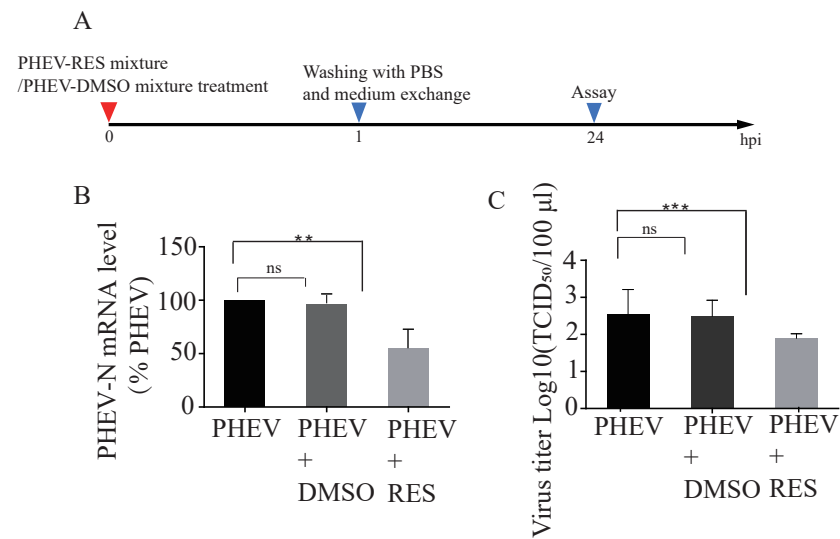

Supplement: Supplementary file 1 — Additional file 1: N2a cells infected with PHEV were treated with RES Virus inactivation assay. A Schematic diagrams illustrating the experimental design for time-of-addition experiments. B Expression of PHEV mRNA in N2a cells at 24 h as determined by qPCR assay. PHEV mRNA levels were quantified and normalized to GAPDH levels, respectively. C TCID50 values were the means of three repeated titrations at the time points indicated. Data are presented as means ± SEM. *p < 0.05, **p < 0.01, ***p < 0.001 based on comparisons to untreated PHEV-infected cells. [file 12985_2022_1953_MOESM1_ESM.pdf]

## Additional file 2

Mock

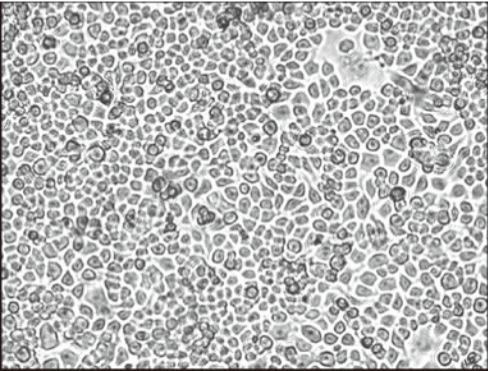

RES+PHEV

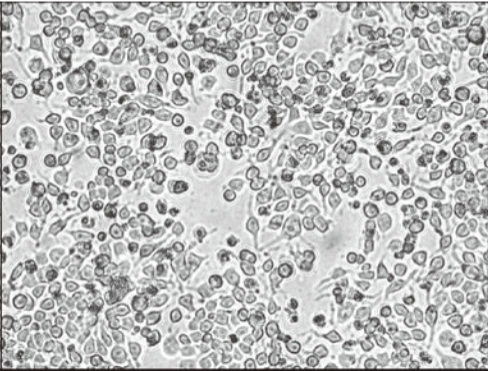

DMSO+PHEV

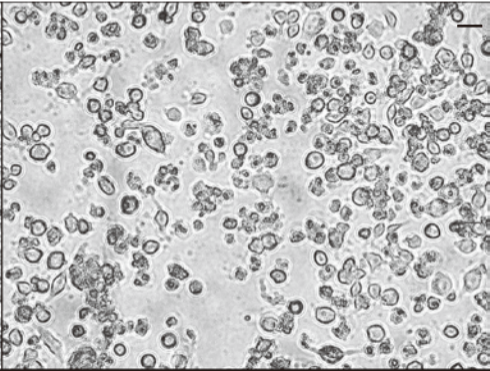

Supplement: Supplementary file 2 — Additional file 2: N2a cells were pre-treated with 10 μM RES for 12 h at 37 °C with 5% CO2 before viral adsorption. Next, the culture medium containing the drug was removed then cells were inoculated with PHEV at 100 TCID50 (106.125) for 72 h at 37 °C with 5% CO2. Mock-infected cells stuck tightly to the plate and remained in good condition throughout the experiment. In contrast, RES treatment effectively blocked CPE in the cell cultures compared to DMSO-treated PHEV-infected cells. A Mock-infected cells. B RES-treated PHEV-infected cells. C DMSO-treated PHEV-infected cells. Cell images were captured by a light microscope. The experiment was performed in triplicate and repeated 3 times. Scale bar = 20 μm. [file 12985_2022_1953_MOESM2_ESM.pdf]
